# Lung Resection for Non-Small Cell Lung Cancer following Bronchoscopic Lung Volume Reduction for Heterogenous Emphysema

**DOI:** 10.3390/cancers16030605

**Published:** 2024-01-31

**Authors:** Alfonso Fiorelli, Beatrice Leonardi, Gaetana Messina, Luca Luzzi, Piero Paladini, Chiara Catelli, Fabrizio Minervini, Peter Kestenholz, Leonardo Teodonio, Antonio D’Andrilli, Erino Angelo Rendina, Giovanni Natale

**Affiliations:** 1 Division of Thoracic Surgery, University of Campania Luigi Vanvitelli, 80131 Naples, Italy; beatriceleonardi01@gmail.com (B.L.); adamessina@virgilio.it (G.M.); dott.natale.giovanni@gmail.com (G.N.); 2 Lung Transplantation Unit, University of Siena, 53100 Siena, Italy; luca.luzzi@unisi.it (L.L.); piero.paladini@unisi.it (P.P.); chiara.catelli1992@gmail.com (C.C.); 3 Division of Thoracic Surgery, Cantonal Hospital Lucerne, 6000 Lucerne, Switzerland; fabrizio.minervini@luks.ch (F.M.); peter.kestenholz@luks.ch (P.K.); 4 Division of Thoracic Surgery, Sapienza University, Sant’Andrea Hospital, 00189 Rome, Italy; leonardo.teodonio@uniroma1.it (L.T.); adandrilli@hotmail.com (A.D.); erinoangelo.rendina@uniroma1.it (E.A.R.)

**Keywords:** emphysema, lung cancer, bronchoscopic lung volume reduction, minimally invasive surgery

## Abstract

**Simple Summary:**

In patients with pulmonary emphysema and NSCLC, poor respiratory function may contraindicate surgical resection. Bronchoscopic lung volume reduction (BLVR) is a minimally invasive procedure for management of emphysema associated with improvement in pulmonary function and quality of life. Therefore, we aimed to evaluate the feasibility of lung resection for NSCLC after BLVR. The improvement in pulmonary function obtained with BLVR may allow nonsurgical candidates to undergo lung resection for lung cancer.

**Abstract:**

Bronchoscopic lung volume reduction (BLVR) is a minimally invasive treatment for emphysema. Lung cancer may be associated with emphysema due to common risk factors. Thus, a growing number of patients undergoing BLVR may develop lung cancer. Herein, we evaluated the effects of lung resection for non-small cell lung cancer in patients undergoing BLVR. The clinical data of patients undergoing BLVR followed by lung resection for NSCLC were retrospectively reviewed. For each patient, surgical and oncological outcomes were recorded to define the effects of this strategy. Eight patients were included in our series. In all cases but one, emphysema was localized within upper lobes; the tumor was detected during routine follow-up following BLVR and it did not involve the treated lobe. The comparison of pre- and post-BLVR data showed a significant improvement in FEV1 (29.7 ± 4.9 vs. 33.7 ± 6.7, *p* = 0.01); in FVC (28.5 ± 6.6 vs. 32.4 ± 6.1, *p* = 0.01); in DLCO (31.5 ± 4.9 vs. 38.7 ± 5.7, *p* = 0.02); in 6MWT (237 ± 14 m vs. 271 ± 15 m, *p* = 0.01); and a reduction in RV (198 ± 11 vs. 143 ± 9.8, *p* = 0.01). Surgical resection of lung cancer included wedge resection (*n* = 6); lobectomy (*n* = 1); and segmentectomy (*n* = 1). No major complications were observed and the comparison of pre- and post-operative data showed no significant reduction in FEV1% (33.7 ± 6.7 vs. 31.5 ± 5.3; *p* = 0.15) and in DLCO (38.7 ± 5.7 vs. 36.1 ± 5.4; *p* = 0.15). Median survival was 35 months and no cancer relapses were observed. The improved lung function obtained with BLVR allowed nonsurgical candidates to undergo lung resection for lung cancer.

## 1. Introduction

Lung cancer is the main cause of death worldwide. Non-small cell lung cancer (NSCLC) accounts for 80% of all cases [1]. The recommended treatment for early-stage NSCLC has historically been lung resection with mediastinal lymph node dissection. Despite the advancements in radiation therapy, chemotherapy, and targeted drug therapy, it remains the best treatment according to current National Comprehensive Cancer Network (NCCN) guidelines [2,3]. Resected patients with stage I disease have 5-year overall survival rates of more than 70%, and outcomes are even better for patients with tumor size less than or equal to 2 cm [4]. Due to common risk factors such as smoking, lung cancer may be associated with pulmonary emphysema, but the poor respiratory function related to emphysema may preclude lung resection.

In the last decades, bronchoscopic lung volume reduction (BLVR) has been emerging as a minimally invasive procedure for management of emphysema [5,6,7,8,9]. The lobar deflation obtained after BLVR produced significant improvements in pulmonary function, in the 6-min-walk test, and in quality of life, and these benefits remained still evident up to 5 years from the treatment [10,11,12]. Considering the growing number of patients undergoing BLVR, lung cancer may be associated with BLVR, but the management of lung cancer in this subset of patients is still uncertain.

The aim of this study was to evaluate the effects of lung resection surgery in patients suffering from heterogeneous emphysema and lung cancer who were previously subjected to BLVR.

## 2. Materials and Methods

### 2.1. Study Design

This was a multicenter retrospective study. The clinical data of patients suffering from heterogenous emphysema and lung cancer and underwent BLVR followed by lung resection for NSCLC were retrospectively reviewed. For each patient, surgical and oncological outcomes were recorded to define the effects of surgery.

The study design was approved by the Local Ethics Committee of University of Campania Luigi Vanvitelli, the coordinator center of the study, and then by the Local Ethics Committee of each participating center. Due to the retrospective nature of the study, no specific approval code was required because there was no modification in the standard patient care. In all cases, informed signed written consent was obtained from the patients for the treatment, and they were aware that their data were used anonymously for scientific purposes only.

### 2.2. Bronchoscopic Lung Volume Reduction Evaluation

**Figure 1:**
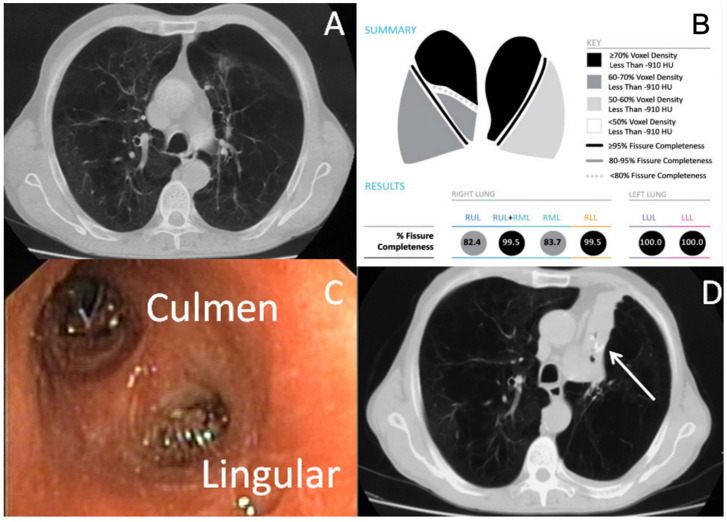
BLVR in a patient with heterogenous emphysema of LUL. (**A**): Computed Tomography scan shows heterogenous emphysema of upper lobes; (**B**): Quantitative computed tomography analysis shows complete fissure integrity between left upper lobe and left lower lobe; (**C**): Two valves were inserted to occlude the culmen and the lingular bronchus of the left upper lobe; (**D**): Complete collapse of left upper lobe was obtained after valve insertion (white arrow: endobronchial valve and collapsed left upper lobe).

Before and after the procedure, all patients underwent standard pulmonary function tests (PFTs), including forced expiratory volume in one second (FEV1), residual volume (RV), diffusing capacity of the lung for carbon monoxide (DLCO), 6-minute-walk test (6MWT), and PaO_2_ and PaCO_2_ (measured at rest while breathing room air). The data were presented as a percentage of predicted values and performed according to the American Thoracic Society and the European Respiratory Society (ATS/ERS) guidelines [13]. Patients were scheduled for BLVR based on the criteria from the Endobronchial Valve for Emphysema Palliation Trial (VENT study) [14] as follows: (i) age of 40–75 years; (ii) FEV1 < 45% of the predicted value; (iii) TLV > 100% of the predicted value; (iv) RV > 150% of the predicted value; (v) PaCO_2_ < 50 mmHg; (vi) PaO_2_ > 45 mmHg, and (vii) 6MWT ≥ 140 m. High-resolution computed tomography (HRCT) scans and lung perfusion scans identified the most affected target lobe. Collateral ventilation (CV) was directly measured by Chartis system (https://pulmonx.com/chartis-tablet-system/) by occluding the target lobe with a dedicated balloon catheter, or indirectly assessed by measuring fissure integrity (FI) using a quantitative CT (QCT) analysis with dedicated software (StratX™ lung analysis platform, https://pulmonx.com/stratx/) that quantified the amount of emphysema and scored FI. As previously reported [15,16,17], patients with a grade of CT-fissure integrity ≥ 95% underwent BLVR with valves, whereas in patients with fissure integrity between 75% and 90%, Chartis assessment was performed to confirm the lack of CV before proceeding with valve implant. Patients with fissure integrity < 75% on QCT analysis or those with fissure integrative > 75% but CV-positive after Chartis assessment were excluded from BLVR with one-way valves and scheduled for other treatments, such as BLVR with coils. In all cases, the aim of BLVR treatment was to obtain the collapse of the most emphysematous lobe. An example is reported in Figure 1.

### 2.3. Lung Cancer

Before the operation, all patients underwent standard cardio-respiratory evaluation and oncological staging through total-body computed tomography/positron emission tomography scan (CT/PET). Based on the last version of the European Society of Thoracic Surgeons (ESTS) guidelines [18], in patients with CT-enlarged or PET-positive mediastinal lymph nodes (cN2 patients), patients with central tumors or those with clinical pathological N1 nodes (cN1), or patients with tumor > 30 mm, pre-operative mediastinal staging was indicated via cervical mediastinoscopy, endobronchial ultrasound (EBUS)-guided transbronchial needle aspiration (TBNA), thoracoscopy, or Chamberlain incision, decided by the participating centers. The type of resection (lobar and/or sublobar), the surgical strategy (thoracoscopy, robotic surgery, or thoracotomy), and the use of glue or synthetic materials to cover the resection line were decided by the participating centers. In all patients, systematic lymph node resection was carried out for lung cancer staging and to guide future treatment. Operative, peri-operative, and post-operative complications, post-operative function, tumor recurrence, survival, and cause of death were registered for each patient. Based on the Clavien–Dindo classification [19], each complication was graded from I (adverse effect that altered the standard post-operative course without requiring specific treatment) to V (adverse event that led to death), reflecting increasing severity and complexity of management regardless of the type of complication.

### 2.4. Statistical Analysis

Data were summarized as mean and standard deviation (SD) for normally distributed continuous variables, or absolute number and percentage for categorical variables. Differences between baseline and post-treatment data were compared using the Chi-square test for categorical variables and *t*-test for continuous variables. A *p*-value < 0.05 was considered significant. MedCalc statistical software (Version 12.3, Broekstraat 52; 9030 Mariakerke, Belgium) was used for the analysis.

## 3. Results

Eight patients were included in our series; their characteristics are summarized in Table 1. The main age was 64 ± 2.3 years old and all patients were male. All patients presented severe heterogeneous emphysema associated with cardiac disease in three cases, renal disease in one case and diabetes in one case. The emphysema was localized in all cases but one (patient 2) within the upper lobe. In eight cases, the tumor was detected during routine CT follow-up at 21.7 ± 9.5 months following BLVR procedure, while in only one case (patient 3) the diagnosis of tumor was concomitant to the BLVR procedure. In only one case (patient 4), the tumor involved the treated lobe. In three cases (patient 3, patient 4, and patient 6) a pre-operative diagnosis of tumor was obtained after CT-guided biopsy, while the other five patients underwent upfront surgical resection due to the high suspicion of malignancy by PET-CT scan findings. Seven patients presented a peripheral tumor ≤ 30 mm and without enlarged lymph nodes on CT and pathological uptake on PET (cN0), thus mediastinal invasive staging was not indicated. Only patient 2 underwent pre-operative mediastinal staging by EBUS-TBNA as the tumor was ≥30 mm. The EBUS-TBNA results were negative for mediastinal involvement and the patient underwent lung resection of the tumor. In all cases but one (patient 7), the histological diagnosis was adenocarcinoma (pathological stage I).

### 3.1. Bronchoscopic Lung Volume Reduction

The data are summarized in Table 2. All patients had a diagnosis of severe emphysema (five patients had GOLD stage III and three patients presented GOLD stage IV) and a residual volume (RV) ≥ 150% predicted. BLVR were performed in six cases by one-way valves and in two cases (patient 1 and patient 2) by coils. In all cases, collapse of the treated lobe was obtained after the procedure. Only one patient presented pneumothorax (patient 6) after BLVR, which was successfully treated with insertion of chest drainage and resolved 5 days later. A significant improvement in FEV1 (29.7 ± 4.9 vs. 33.7 ± 6.7, *p* = 0.01); in forced vital capacity (FVC) (28.5 ± 6.6 vs. 32.4 ± 6.1, *p* = 0.01); in DLCO (31.5 ± 4.9 vs. 38.7 ± 5.7, *p* = 0.02); in 6MWT (237 ± 14 m vs. 271 ± 15 m, *p* = 0.01); and a reduction in RV (198 ± 11 vs. 143 ± 9.8, *p* = 0.01) was observed after BLVR.

### 3.2. Tumor Resection

**Figure 2:**
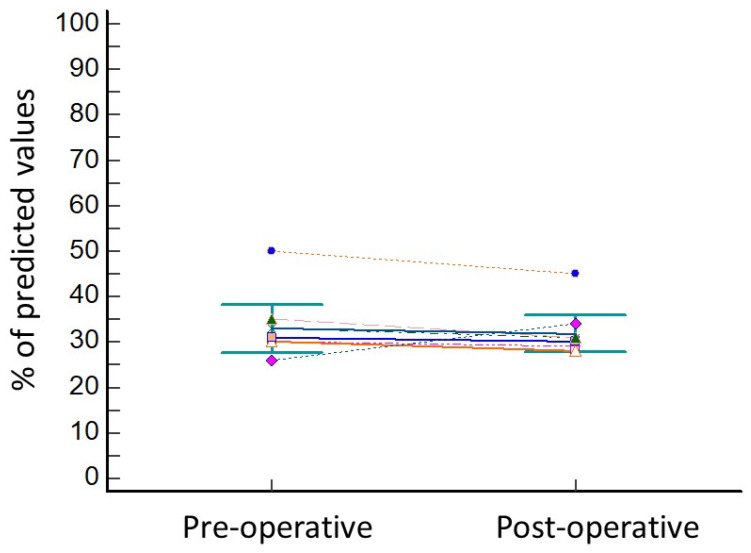
Variation in FEV1 before and after lung resection for cancer. Every different coloured line represents the variation in FEV1 values of one of the patients of the series.

**Figure 3:**
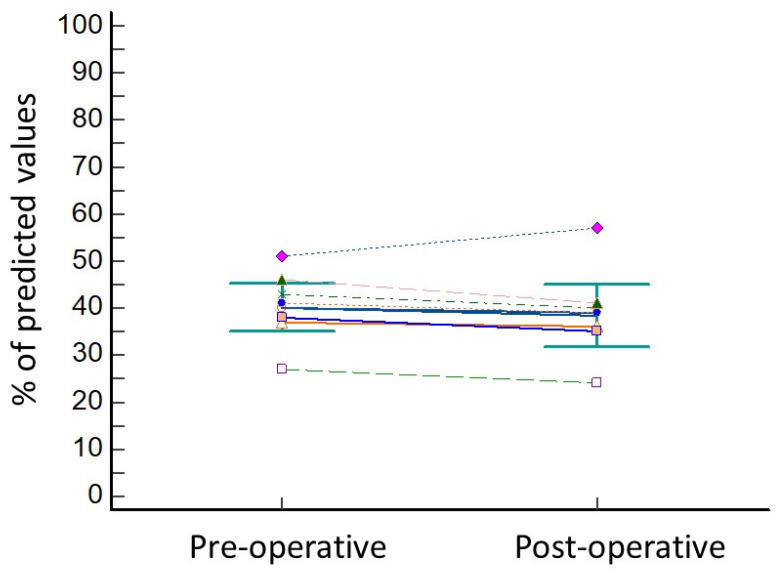
Variation inf DLCO before and after lung resection for cancer. Every different coloured line represents the variation in DLCO values of one of the patients of the series.

The lung resection of the tumor was performed by standard three-portal thoracoscopy in five cases, robotic surgery in one case (patient 8), while in two cases (patient 4 and patient 6) a conversion to thoracotomy from thoracoscopy was needed due to the presence of tenacious adhesions. In all cases, operative view showed the collapse of the treated lobe. The wedge resection of right segment 3 was performed in three cases, of right segment 4 in one case, of left segment 9 in one patient, and of left segment 10 in one patient. One patient underwent lobectomy of the right upper lobe (patient 4) and another patient (patient 3) underwent segmentectomy of right segment 6. No intra-operative complications were observed, and the length of hospital stay was 17 ± 6.9 days. The post-operative complications included minor complications, as persistent air leaks (PAL) were observed in three cases (patient 3, patient 4, and patient 8) and atrial fibrillation occurred in one case (patient 2). Atrial fibrillation was resolved with medical therapy (Grade 2) 7 days after operation, while air leaks spontaneously stopped without requiring endoscopic or surgical treatments (Grade 2). The comparison of pre-operative versus post-operative data showed no significant reduction in FEV1% (33.7 ± 6.7 vs. 31.5 ± 5.3; *p* = 0.15; Figure 2) and in DLCO% (38.7 ± 5.7 vs. 36.1 ± 5.4; *p* = 0.15; Figure 3).

The median survival was 35 months. Three patients died, from cardiac disease in two cases (patient 6 and patient 7) and ischemic attack in one case (patient 4).

## 4. Discussion

Emphysema is a leading cause of morbidity and mortality. It is characterized by air trapping and hyperinflation due to the destruction of the lung parenchyma, loss of elastic recoil, and collapse of the small airways [5,6,7,8,9,10,11]. Patients with advanced emphysema are usually non-responders to medical treatment. Lung transplantation and/or LVRS are the main treatments for these patients. However, lung transplantation has limited availability due to strict selection criteria and suitability of donor organs [20]. Lung volume reduction surgery (LVRS) through the resection of the emphysematous parenchyma reduces the mismatch in size between the hyperinflated lungs and chest cavity and restores respiration muscle activity. However, the NETT [21] trial found a post-operative mortality and morbidity of 6% and 30%, respectively. The most common major complications were cardiac arrythmia and pneumonia observed in 23.5% and 18% of patients, respectively, and 21.8% of patients needed reintubation. Tracheostomy was performed in 8.2% of patients, and 5.1% of patients were not weaned from mechanical ventilation. Higher age, lower value of FEV1, and the use of steroids were the main risk factors for post-operative morbidity and mortality. Air leaks occurred in 90% of patients, and they persisted for 30 days in 12% of patients.

Thus, less invasive procedures have been proposed for management of emphysema. In the last two decades, BLVR has emerged as an alternative to LVRS for management of emphysema due lower morbidity and mortality [5]. Endobronchial valves, coils, and thermal vapor ablation are the main strategies for BLVR. Endobronchial one-way valves are the most common strategy reported in the literature for obtaining BLVR [22]. The valves are inserted through fibreoptic bronchoscopy in the segmental bronchi in a targeted lobe. Valves allow the expiratory flow of air while blocking inspiratory flow in the targeted lobe. This mechanism allows the collapse of the lobe, resulting in a reduction in lobar volume. All studies [5,6,7,8,9,10,11,22] showed that the collapse of the treated lobe was associated with the improvement in respiratory function. The presence of collateral ventilation and the incomplete treatment of a targeted lobe related to nonoptimal placement of the valves were associated with poor results. Thus, only patients with the absence of collateral ventilation should be scheduled for BLVR with endobronchial valves. Collateral ventilation is assessed by indirect or direct methods, as in the present paper. Indirect methods are based on the evaluation of fissure integrity by qualitative or quantitative CT scan, while the direct method consists of endobronchial measurement of collateral ventilation using the Chartis system (PulmonX). Conversely, coil insertion and/or vapor ablation do not require the lack of CV, extending BLVR also to CV-positive patients [22]. Endobronchial coils are shape-memory nitinol devices that are inserted using fiber-bronchoscopy in the sub-segmental airways under fluoroscopic guidance. After deployment, the coil retains its non-straight predominant shape, leading to compression of the diseased parts of the lung and targeting lobar volume reduction. Thermal vapor ablation consists of the endoscopic instillation of heated water in the most emphysematous lobe. It induces the local inflammatory response, resulting in parenchymal fibrosis and lung volume reduction. Unlike valves, these strategies are not fully reversible and they are reviewed for CV-positive patients who are not suitable for valve treatment [22].

As smoking is the most common risk factor for developing not only lung cancer but also chronic obstructive pulmonary disease, lung cancer and emphysema can coexist. However, no papers investigated the effects of surgery in patients suffering from pulmonary emphysema and lung cancer and were previously subjected to BLVR. Considering the extreme rarity of this clinical scenario, we planned a multicenter study including all patients in whom lung cancer was surgically treated following BLVR. Our study population included eight consecutive patients. BLVR was performed in six cases by one-way valves and in two cases (patient 1 and patient 2) by coils due to the presence of CV.

In seven patients, lung cancer was detected during routine CT follow-up after BLVR, while in one case, BLVR was concomitant to lung resection. The patient underwent BLVR of the right upper lobe and, 2 months later, anatomical resection of the apical segment of the right lower lobe was performed. Several studies showed that emphysematous patients had a smaller reduction in post-operative function than that expected after lung resection for cancer and, in some cases, respiratory function was also improved [23,24,25,26,27,28]. Carretta et al. [24] retrospectively evaluated the clinical and radiological data of 35 patients affected by emphysema who underwent lung resection for NSCLC. Upper lobectomy was performed in 25 patients, lower lobectomy in 7 patients, and middle lobectomy in 3 cases. A significant correlation was found between the improvement in post-operative FEV1 and the severity of the emphysema scored by CT scan. McKenna et al. [25] reported concomitant lung resection for stage I NSCLC and LVRS in 11 patients affected by severe emphysema. In seven patients, wedge resection was performed, while four patients underwent lobectomy. No intra-operative and post-operative major complications were observed. After operation, an improvement in FEV1 was observed compared to the pre-operative value (49% vs. 21.7%). Choong et al. [26] reviewed the clinical data of 21 patients with lung cancer and concomitant severe emphysema. They underwent a combined operation aimed at resecting the lung cancer and reducing the emphysema. No post-operative deaths were observed. In all cases, a significant improvement in post-operative lung function was observed. The 1-year and 5-year survival rates were 100% and 62.7%, respectively. Edwards et al. [27] performed lobectomy for management of lung cancer in 14 patients with severe emphysema. The patients had ppoFEV1 less than 40%, which represented the lower limit of acceptable risk based on the current guidelines [29,30]. Despite all, in all of these cases, no significant changes in respiratory function were found before and after operation. Additionally, the 1-year post-operative survival rate of these frail patients was similar to those with better pre-operative lung function. Thus, the authors concluded that the cut-off point of 40% for ppoFEV1 should not be clinically binding and current guidelines [29,30] should include an acknowledgement of the effects of surgery in heterogenous emphysema.

However, these beneficial results were evident when the tumor involved the emphysematous lobe, as complete lobectomy achieved the dual function of lung resection for cancer and LVRS. By contrast, in our case, the tumor did not involve the emphysematous lung and the lung resection for cancer was associated with the resection of the functioning parenchyma, resulting in significant reduction in post-operative pulmonary function. Thus, our patient was considered unfit for upfront surgical resection and scheduled for BLVR. The beneficial effects of BLVR reduced the negative effect of lung resection for cancer and allowed performing surgical treatment, which would otherwise not have been indicated because of limited pulmonary function. Similarly, Perikleous et al. [31] reported a ‘hybrid bilobectomy’ in an emphysematous patient with lung cancer of the middle lobe. The procedure consisted of BLVR of the right lower lobe followed by thoracoscopic middle lobectomy.

From a clinical point of view, the improvement in respiratory function obtained with BLVR allowed nonsurgical candidates to undergo lung resection for cancer. In all patients, the atelectasis of the treated lobe obtained with BLVR switched the air to more perfused parenchyma, allowed the re-expansion of the previously compressed alveoli, and facilitated the ventilation and function of the relatively healthier lung. In addition, the reduction in emphysematous parenchyma improved the diaphragm movement and the work of breathing. In the VENT study [14], lobar atelectasis after valve insertion was associated with significant improvement in both FEV1 and 6MWT compared to patients in whom no lobar atelectasis was obtained after valve insertion. Similarly, Garzon et al. [32] reported the upfront resection of a lung nodule via thoracoscopy within the left upper lobe in a patient who previously underwent bilateral BLVR of both lower lobes. The pathological results diagnosed a granulomatous lesion without evidence of malignancy. Yet, also in this case, the improvement in lung function obtained with BLVR allowed surgical resection of a lung lesion in a previously nonsurgical candidate. By contrast, Tummino et al. [33] reported the case of a patient who developed lung cancer in the treated lobe 1 year after BLVR. In this case, BLVR was not associated with an improvement in respiratory function. The patient was considered unfit for lung resection and underwent chemotherapy. He died 2 months later of septicemia related to chemotherapy.

From a technical point of view, the operation was performed by minimally invasive surgery in six cases, including thoracoscopic and robotic surgery. In two cases, the conversion to thoracotomy from thoracoscopy was needed due to the presence of dense adhesions. However, in all cases, the atelectasis of the treated lobe did not complicate the surgical resection of tumor. Yet, Perikelus et al. [31] reported that the atelectasis of the ipsilateral lobe could facilitate the identification of fissure during anatomical resection and prevented post-operative air leaks. Furthermore, the stapled fissure reduced the risk of CV, improving the results of BLVR. In our study population, two patients underwent anatomical resection of the lung cancer including segmentectomy (patient 3) and lobectomy (patient 4), while in the other six cases, wedge resection of the tumor was performed as these patients presented a ppoFEV1 less than 35%. Resection of the tumor following BLVR was a safe procedure and did not have a significant impact on respiratory function, as demonstrated by the lack of significant differences between pre-operative and post-operative respiratory data. In fact, in only one patient was a lobectomy of the non-functioning parenchyma performed as it was the same lobe treated with BLVR. Furthermore, considering the early stage of lung cancer, this strategy was also oncologically indicated. The median survival was 35 months; three patients died during the follow-up, but in all patients, no tumor recurrence was observed. Our results were in line with the results of several metanalyses [34,35,36] showing that intentional sublobar resection was similar in survival to lobectomy for the treatment of stage I NSCLC ≤ 2 cm. Furthermore, Altorki et al. [37], in a prospective randomized multicenter study, compared 340 patients who underwent sublobar resection versus 357 patients who underwent lobar resection for management of T1aN0 NSCLC. The median follow-up was 7 years. The overall survival and disease-free survival were similar between the two study groups. Yet, the incidence of loco-regional recurrence and distal recurrence rates were not different between the two study groups. Post-operative morbidity included minor complications (Grade 2), as persistent air leaks were observed in three cases and atrial fibrillation occurred in one case. Atrial fibrillation resolved with medical therapy 7 days after operation while air leaks spontaneously stopped without requiring endoscopic or surgical treatments. These complications were likely associated with the underlying emphysematous disease. Sato et al. [38] compared the surgical outcomes of 364 patients undergoing lung resection for management of stage I NSCLC in relation to the grade of emphysema in before and after matching analysis. They found that the presence of emphysema affected survival and was associated with the development of post-operative pneumonia and supraventricular tachycardia. The incidence of supraventricular tachycardia (SVT) following lung resection ranged between 10 and 28%, and SVT developed more frequently in patients with emphysema [38]. The mechanism is still not defined. In theory, emphysematous patients present pulmonary hypertension not only at rest but also during exercise compared to normal subjects. Thus, right heart stress following lung resection could occur with an increase in pulmonary artery pressure, resulting in higher incidence of SVT in emphysematous patients. PAL was a major complication of pulmonary resection, with an incidence ranging from 5% to 25% [39,40]. Emphysema is a potential prognostic factor leading to PAL due to extreme parenchymal fragility and incomplete or delayed lung re-expansion. Intra-operative strategies such as buttressed staplers, the fissure-less technique, pleural tenting, glues, and lung sealants might prevent the development of PAL. In our series, there was no standard strategy for the intra-operative management of air leaks and it could concur with the different incidence of PAL in our series.

This study underlines several messages for physicians. Considering the growing number of emphysematous patients undergoing BLVR, these patients should be included in lung cancer screening programs to allow for early detection of lung cancer. BLVR could significantly improve pulmonary function, allowing previously nonsurgical candidates to undergo lung resection. By contrast, if patients remain unfit for lung resection of the tumor after BLVR, they could be reviewed for alternative treatment, such as stereotactic body radiotherapy, considering the early diagnosis of lung cancer. 

The small number of patients, the lack of a control group, and the different procedures for obtaining BLVR and for resecting lung cancer due to the lack of standardized protocols were the main limitations of this study.

## 5. Conclusions

Lung resection for cancer seems to be a valuable and safe procedure in patients with heterogeneous emphysema previously subjected to BLVR, but our preliminary results should be confirmed by further and larger experiences and also in randomized studies.

## Figures and Tables

**Table 1:** Patient characteristics.

Pt	Age	Comorbidity	Site of BLVR	BLVR to Cancer Resection Time (Months)	Site of Cancer	Resection	Histology	Stage	Complications
1	65	Emphysema Cardiac Renal	RUL	22	ML	Wedge of S4	Adenoca.	T1N0M0	None
2	57	Emphysema Cardiac	RLL	30	RUL	Wedge of S3	Adenoca.	T2aN0M0	Atrial fibrillation
3	58	Emphysema	RUL	2	RLL	Segmentectomy (S6)	Adenoca.	T1N0M0	PAL
4	70	Emphysema	RUL	24	RUL	Lobectomy	Adenoca.	T1N0M0	PAL
5	69	Emphysema	LUL	26	LLL	Wedge of S9	Adenoca.	T1N0M0	None
6	71	Emphysema Cardiac	LUL	6	RLL	Wedge of S10	Adenoca.	T1N0M0	None
7	69	Emphysema	LUL	12	RUL	Wedge of S3	Squamous ca.	T1N0M0	None
8	59	Emphysema, diabetes	LUL	32	RUL	Wedge of S3	Adenoca.	T1N0M0	PAL

Abbreviations: Pt: patient; RUL: right upper lobe; ML: middle lobe; RLL: right lower lobe; LLL: left lower lobe; PAL: persistent air leak; Adenoca.: adenocarcinoma; ca.: carcinoma; BLVR: bronchoscopic lung volume reduction; S: segment.

**Table 2:** Functional data before and after BLVR.

Variables	Pre-BLVR	Post-BLVR	*p*-Value
O_2_ saturation %	92 ± 6.1	92 ± 5.3	0.57
pO_2_	68.6 ± 12	69.5 ± 13	0.66
pCO_2_	43.4 ± 3.9	42.7 ± 7.5	0.34
FEV1%	29.7 ± 4.9	33.7 ± 6.7	0.01
FVC%	28.5 ± 6.6	32.4 ± 6.1	0.01
RV%	198 ± 11	143 ± 9.8	0.003
6MWT	237 ± 14	271 ± 15	0.01
DLCO%	31.5 ± 4.9	38.7 ± 5.7	0.02

Abbreviations: FEV1: forced expiratory volume in one second; FVC: forced vital capacity; RV: residual volume; 6MWT: 6-minute-walk test; DLCO: diffusing capacity of the lung for carbon monoxide.

## Data Availability

The data presented in this study are available upon request from the corresponding author.
